# Anomalous Nernst effect in stressed magnetostrictive film grown onto flexible substrate

**DOI:** 10.1038/s41598-019-51971-7

**Published:** 2019-10-25

**Authors:** Acácio Silveira Melo, Alexandre Barbosa de Oliveira, Carlos Chesman, Rafael Domingues Della Pace, Felipe Bohn, Marcio Assolin Correa

**Affiliations:** Departamento de Física, Universidade Federal do Rio Grande do Norte, 59078-900 Natal, RN Brazil

**Keywords:** Materials science, Nanoscience and technology

## Abstract

The anomalous Nernst effect in nanostructured magnetic materials is a key phenomenon to optimally control and employ the internal energy dissipated in electronic devices, being dependent on, for instance, the magnetic anisotropy of the active element. Thereby, here, we report a theoretical and experimental investigation of the magnetic properties and anomalous Nernst effect in a flexible magnetostrictive film with induced uniaxial magnetic anisotropy and under external stress. Specifically, we calculate the magnetization behavior and the thermoelectric voltage response from a theoretical approach for a planar geometry, with magnetic free energy density that takes into account the induced uniaxial and magnetoelastic anisotropy contributions. Experimentally, we verify modifications of the effective magnetic anisotropy by changing the external stress, and explore the anomalous Nernst effect, a powerful tool to investigate the magnetic properties of magnetostrictive materials. We find quantitative agreement between experiment and numerical calculations, thus elucidating the magnetic behavior and thermoelectric voltage response. Besides, we provide evidence to confirm the validity of the theoretical approach to describe the magnetic properties and anomalous Nernst effect in ferromagnetic magnetostrictive films having uniaxial magnetic anisotropy and submitted to external stress. Hence, the results place flexible magnetostrictive systems as promising candidates for active elements in functionalized touch electronic devices.

## Introduction

New and more efficient ways to make use the internal energy converted into heat in electronic devices are of crucial importance for a sustainable future^1,2^. In this field, the engineering of magnetostrictive films onto flexible substrates is an exciting area of research with potential to the development of energy-efficient technologies, covering the impacts of the external stress on the quasi-static and dynamic magnetic properties^3–6^. Specifically, the interplay of magnetostrictive and temperature effects provides us a playground to the investigation of fundamental anisotropic magnetothermoelectric effects, for instance, the spin polarization dependence of the thermoelectric energy^7–10^.

In magnetic materials, the thermoelectric voltage, defined as the direct conversion of the temperature gradient to electrical voltage, is investigated through thermomagnetic phenomena, such as the spin Seebeck effect (SSE) and the anomalous Nernst effect (ANE)^11,12^. Both of them essentially consist in the application of a magnetic field and a temperature gradient, thus generating an electric field $\overrightarrow{E}$. Specifically, for the anomalous Nernst effect in nanostructured magnetic materials, an electric field ${\overrightarrow{E}}_{ANE}$ is induced by the interplay of the magnetization $\overrightarrow{m}$ and the temperature gradient ∇*T*, being a result of ${\overrightarrow{E}}_{ANE}\propto \overrightarrow{m}\times \nabla T$. Thus, given that the corresponding thermoelectric voltage *V*_*ANE*_ in magnetic materials is observable even with small thermal gradients at room temperature, ANE becomes a key effect to optimally control and employ the internal energy dissipated in electronic devices. In the near past, ANE has been extensively investigated, disclosing that *V*_*ANE*_ is strongly dependent on the crystallographic orientation in magnetic materials^13^, type of material (for instance, Fe and Ni have ANE coefficient with opposite signs), thickness of the layers in the film geometry^12,14^, and magnetic anisotropy^15^. However, despite the recent advances in this field, there still are several aspects related to the anomalous Nernst effect in nanostructured ferromagnetic materials that are not yet fully understood or explored. Among the different investigated ferromagnets, materials with high-spin polarization are the main elected to constitute spintronics devices. In this context, CoFeB alloys in the film geometry arise as one of the most promising candidates for this kind of application due to their magnetic properties, such as high permeability, high saturation magnetization, low coercive field, and well-defined magnetic anisotropy^16–19^. These features place the CoFeB alloys suitable to the production of, for instance, magnetic tunnel junctions with high magnetoresistance^20–23^ and spin-transfer torque^23^, as well as to the investigation of a sort of phenomena, as spin pumping^24,25^, spin Hall effect^26^, and inverse spin Seebeck effect^27^. It is known that the magnetic properties of CoFeB films are strongly dependent on the thickness^28–30^. Films thinner than 5 nm usually exhibit strong perpendicular magnetic anisotropy (PMA)^28^, while the thicker ones commonly show in-plane magnetic anisotropy^29,30^. Moreover, due to its magnetostrictive properties, thick-CoFeB films have been grown onto flexible substrates, thus becoming essential systems to investigate the influence of the stress on the effective magnetic anisotropy^16,17^, as well as appearing as promising materials for application in flexible magnetic devices^31^, disposable electronics, smart cards, light-emitting diodes, wearable electronics and a broad range of sensors^18,32–35^.

To pave the way, looking for effective integration between the thermoelectric voltage experiments and flexible spintronic devices, the anomalous Nernst effect emerges as a powerful tool to explore thermoelectric effects in samples with magnetostrictive properties. In this work, we perform a theoretical and experimental investigation of the magnetic properties and anomalous Nernst effect in a flexible magnetostrictive film with induced uniaxial magnetic anisotropy and under external stress. Specifically, from the theoretical approach, we calculate the magnetization behavior and the thermoelectric voltage response; experimentally, we verify modifications of the effective magnetic anisotropy in stressed samples through thermoelectric voltage measurements, and explore the anomalous Nernst effect as an efficient way to study flexible magnetostrictive film by modifying both, the magnetic field and external stress. We find quantitative agreement between experiment and numerical calculations, thus elucidating the magnetic properties and thermoelectric voltage response. Besides, we provide evidence to confirm the validity of the theoretical approach to describe the magnetic properties and anomalous Nernst effect in ferromagnetic magnetostrictive films having uniaxial magnetic anisotropy and submitted to external stress.

## Results

### Theoretical approach

**Figure 1:**
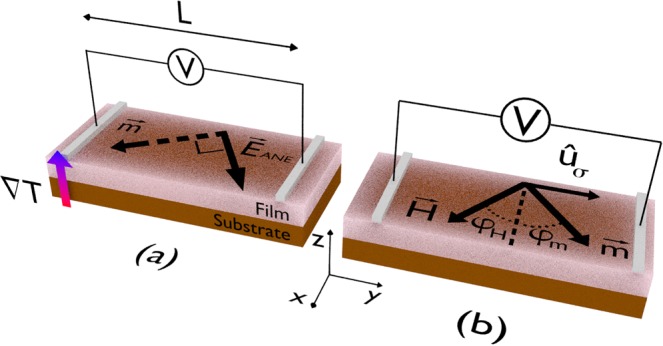
Schematic configuration of our theoretical system–a ferromagnetic magnetostrictive film with uniaxial magnetic anisotropy, which is submitted to external stress. (**a**) Anomalous Nernst effect in a film. In our numerical calculation, while the magnetization $\overrightarrow{m}$ lies in the film plane, the temperature gradient ∇*T* is normal to the plane. The electric field associated with the anomalous Nernst effect is a result of ${\overrightarrow{E}}_{ANE}\propto \overrightarrow{m}\times \nabla T$, and thus the thermoelectric voltage *V*_*ANE*_ is proportional to a component of ${\overrightarrow{E}}_{ANE}$, given that its detection is performed with electrical contacts at the ends of the main axis of the film. (**b**) Definitions of the vectors and angles employed in the numerical calculations of magnetization and thermoelectric voltage. Here, we consider the magnetization vector $\overrightarrow{m}$, whose orientation for each given magnetic field value is set by ${\theta }_{m}$ and ${\varphi }_{m}$, the equilibrium angles with respect to the *z* and *x* axes (this latter is also represented by the dashed line above the film), respectively. In particular, due to the film geometry and system thickness, here ${\theta }_{m}$ is constant and equal to 90°. The magnetic field is kept in the film plane, i.e. ${\theta }_{H}={90}^{\circ }$, although its orientation can be modified by varying *φ*_*H*_ from 0° up to 360°, having as reference the dashed line indicated in the illustration. The unit vector ${\hat{u}}_{k}$, not shown here, is defined by ${\theta }_{k}$ and ${\varphi }_{k}$, and indicates the direction of the uniaxial magnetic anisotropy induced during deposition; ${\hat{u}}_{\sigma }$ is given by ${\theta }_{\sigma }={90}^{\circ }$ and ${\phi }_{\sigma }={90}^{\circ }$, and describes the direction of the magnetoelastic anisotropy induced by the stress applied to the sample during the experiment; and ${\hat{u}}_{eff}$, not shown here too, is described by ${\theta }_{{u}_{eff}}$ and ${\phi }_{{u}_{eff}}$, and represents the orientation of the effective magnetic anisotropy, a result of the competition between the contributions of the induced uniaxial and magnetoelastic anisotropies. The unit vector normal to the film plane, also not shown, is along the *z* direction, i.e. $\hat{n}=\hat{k}$. Finally, for the thermoelectric voltage calculation, the temperature gradient ∇*T* is normal to the film plane. Further, the *V*_*ANE*_ detection is performed with electrical contacts at the ends of the main axis of the film.

Here, we focus on a ferromagnetic magnetostrictive film with uniaxial magnetic anisotropy, which is submitted to external stress; and we model it as a planar system, as illustrated in Fig. 1.

In the anomalous Nernst effect in magnetic materials, an electric field ${\overrightarrow{E}}_{ANE}$ is induced by the interplay of the magnetization $\overrightarrow{m}$ of the sample and a temperature gradient ∇*T*; and the relationship between these quantities can be expressed as

1 $${\overrightarrow{E}}_{ANE}=-\,{S}_{N}(\hat{m}\times \nabla T),$$

where ${S}_{N}={\lambda }_{N}{\mu }_{\circ }{m}_{s}$; *λ*_*N*_ is the anomalous Nernst coefficient, *μ*_o_ is the vacuum magnetic permeability, and *m*_*s*_ is the saturation magnetization of the ferromagnetic alloy, which is oriented along to the unit vector $\hat{m}\mathrm{.}$

In our theoretical approach, we consider a typical ANE experiment in a film, in which the temperature gradient is normal to the film plane, while the magnetization lies in the plane, as depicted in Fig. 1(a). The corresponding thermoelectric voltage *V*_*ANE*_, detected by electrical contacts at the ends of the main axis of the film, is thus given by

2 $${V}_{ANE}=-\,{\int }_{0}^{L}{\overrightarrow{E}}_{ANE}\cdot d\overrightarrow{l},$$

where the integration limits are set by the distance between the electrical contacts, which in our case is *L*. As a result, the measured *V*_*ANE*_ is proportional to the ${\overrightarrow{E}}_{ANE}$ component along to the direction defined by the contacts, as we can see in Fig. 1(a,b).

For films under an out-of-plane temperature gradient, *S*_*N*_ can be experimentally estimated through

3 $${S}_{N}=({V}_{ANE}^{{S}_{max}}\,{t}_{f}))/(L\,\Delta {T}_{f}),$$

where ***t***_*f*_ is the film thickness, *L* is the own distance between the probe electrical contacts in the experiment, $\Delta {T}_{f}$ is the temperature variation across the film (see Methods for details on the $\Delta {T}_{f}$ estimation and its relation with the temperature variation $\Delta T$ measured experimentally across the sample), and ${V}_{ANE}^{{S}_{max}}$ is a very particular maximum *V*_*ANE*_ value that we set from experiment. Specifically, this latter is obtained when the sample is magnetically saturated, with $\overrightarrow{m}$ in the film plane and transverse to the detection direction defined by the electrical contacts; this configuration yields ${\overrightarrow{E}}_{ANE}$ parallel to the direction of the voltage detection, having its highest magnitude. After all, our theoretical approach provides a normalized *V*_*ANE*_ response, which is rescaled using an experimental *S*_*N*_ value (see Methods for details on the *S*_*N*_ estimation) for comparison.

The theoretical system and the definitions of the vectors considered to perform the numerical calculations are depicted in Fig. 1(b). Looking at the thermoelectric voltage at a constant in-plane magnetic field that is high enough to keep the film magnetically saturated, the magnetization follows the orientation of the magnetic field, i.e. ${\phi }_{m}={\phi }_{H}$. As consequence, the angular dependence of *V*_*ANE*_ with the magnetic field is considerable simplified,

4 $${V}_{ANE}={S}_{N}|\nabla T|L\,\cos \,{\phi }_{m}\mathrm{.}$$

On the other hand, it is worth remarking that the amplitude and direction of the magnetization may be changed by the application of a magnetic field $\overrightarrow{H}$ and/or external stress *σ*, thus modifying ${\overrightarrow{E}}_{ANE}$ and, consequently, *V*_*ANE*_. At the non-saturated magnetic regime, the equilibrium angle *φ*_*m*_ of the magnetization is a result of the competition between the applied magnetic field and the magnetic anisotropies of the sample, leading to a complex dependence of the *V*_*ANE*_ with the magnetic field. To investigate the magnetic properties and thermoelectric voltage response of magnetostrictive films with induced uniaxial magnetic anisotropy, we employ a modified Stoner-Wohlfarth model^36^. Here, we consider the magnetic free energy density as

5 $$\xi =-\,\overrightarrow{m}\cdot \overrightarrow{H}+4\pi {m}_{s}^{2}{(\hat{m}\cdot \hat{n})}^{2}-\frac{{H}_{k}}{2{m}_{s}}{(\hat{m}\cdot {\hat{u}}_{k})}^{2}-\frac{3}{2}{\lambda }_{s}\sigma {(\hat{m}\cdot {\hat{u}}_{\sigma })}^{2},$$

where $\overrightarrow{m}$ is the magnetization vector of the ferromagnetic layer, $\hat{m}$ is its corresponding versor, *m*_*s*_ is the saturation magnetization, ${H}_{k}\mathrm{=2}{K}_{u}/{m}_{s}$ is the anisotropy field, where *K*_*u*_ is the induced uniaxial magnetic anisotropy constant, ${\lambda }_{s}$ is the saturation magnetostriction constant, *σ* is the applied stress, and $\hat{n}$ is the versor normal to the film plane. The first term of the magnetic free energy density indicates the Zeeman interaction, the second one is the demagnetizing energy density, and the third term is associated to the induced uniaxial magnetic anisotropy oriented along ${\hat{u}}_{k}$. Finally, the fourth term describes the magnetoelastic energy density for an elastically medium with isotropic magnetostriction. This latter term relates the saturation magnetostriction constant ${\lambda }_{s}$ and the stress *σ* applied to the system, and gives rise to the magnetoelastic anisotropy contribution along ${\hat{u}}_{\sigma }$. In particular, the product between ${\lambda }_{s}$ and *σ* modifies the effective magnetic anisotropy of the sample. While for ${\lambda }_{s}\sigma \mathrm{ > 0}$ a magnetoelastic anisotropy axis is induced along the same direction of the applied stress, for ${\lambda }_{s}\sigma \mathrm{ < 0}$, this magnetoelastic anisotropy axis is oriented perpendicularly to the direction of the stress^36–38^.

From the appropriate magnetic free energy density, a routine for the energy minimization determines the equilibrium angles ${\theta }_{m}$ and ${\phi }_{m}$ of the magnetization at a given magnetic field $\overrightarrow{H}$, and we calculate the normalized magnetization $m/{m}_{s}$ and the thermoelectric voltage *V*_*ANE*_ curves.

**Figure 2:**
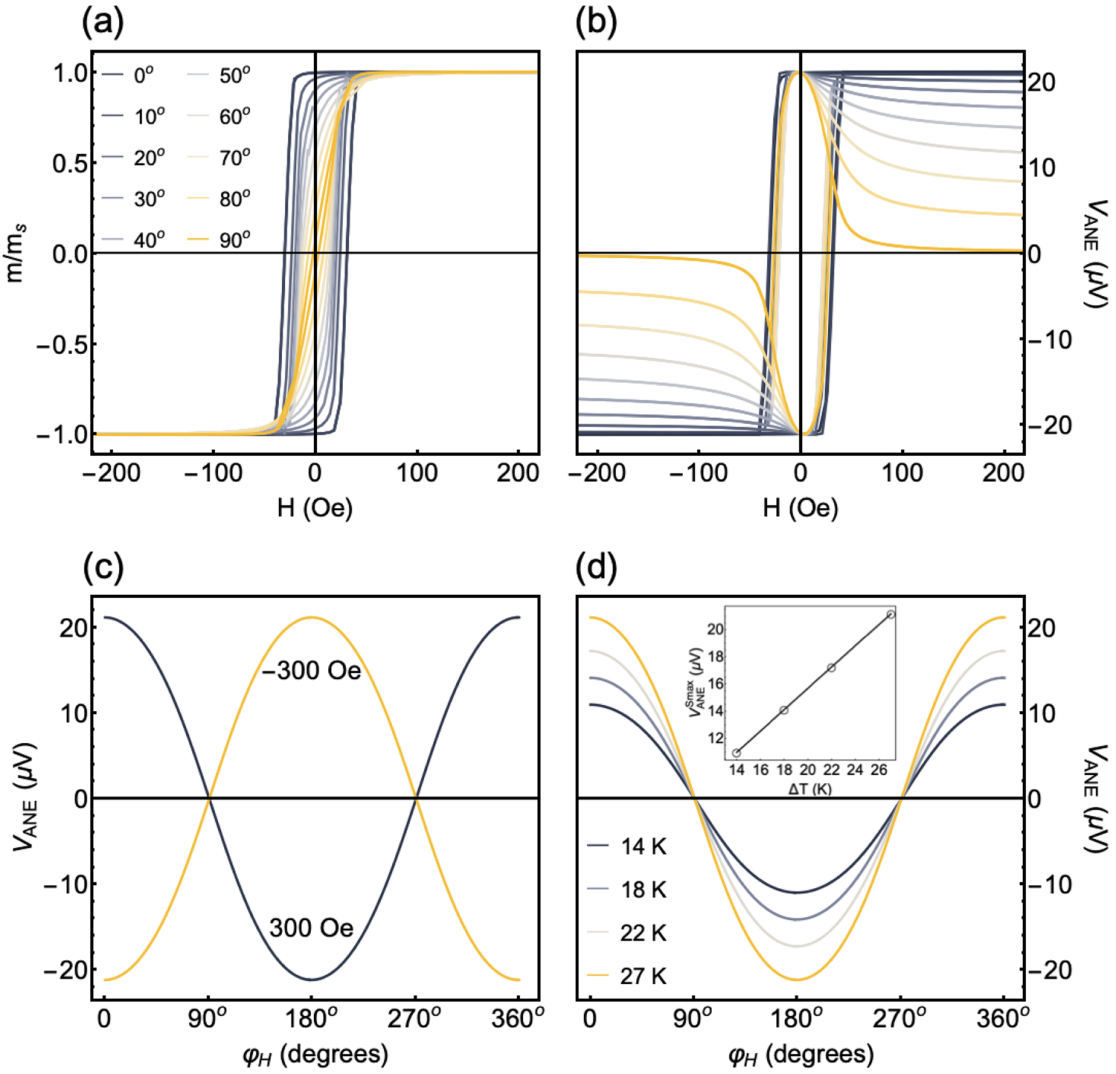
Numerical calculations of the magnetic response and thermoelectric voltage for a film with uniaxial magnetic anisotropy. For the calculations, we consider the following parameters: ${m}_{s}=625$ emu/cm^3^, ${\lambda }_{s}=+\,30.0\times {10}^{-6}$, ${H}_{k}=42$ Oe, ${\theta }_{k}={90}^{\circ }$, ${\phi }_{k}={5}^{\circ }$, ${t}_{f}=300$ nm, $\sigma \mathrm{=0}$ MPa and ${S}_{N}=1.31\times {10}^{-5}$ V/K. For the thermoelectric voltage calculation, the temperature gradient ∇*T* is normal to the film plane. Further, the *V*_*ANE*_ detection is performed with electrical contacts at the ends of the main axis of the film. Notice that this direction is almost transverse to ${\hat{u}}_{k}$, which defines the induced uniaxial magnetic **a**nisotropy. (**a**) Normalized magnetization curves for distinct *φ*_*H*_ values. (**b**) *V*_*ANE*_ response, with $\Delta T=27$ K, as a function of the magnetic field for the very same *φ*_*H*_ values. (**c**) *V*_*ANE*_, at $H=\pm \,300$ Oe and with $\Delta T=27$ K, as a function of *φ*_*H*_. At this field value, our system is magnetically saturated. (**d**) Similar plot for the *V*_*ANE*_ at $H=+\,300$ Oe for distinct Δ*T* values. In the inset, the dependence of the ${V}_{ANE}^{Smax}$ value with Δ*T* is shown.

First of all, to confirm the validity of our theoretical approach, we consider a system consisting of a film with uniaxial magnetic anisotropy and without stress, i.e. $\sigma \mathrm{=0}$ MPa. For the numerical calculations, we take into account the following system parameters: ${m}_{s}=625$ emu/cm^3^ and ${\lambda }_{s}=+\,30.0\times {10}^{-6}$, which are characteristic values of the Co_40_Fe_40_B_20_ alloy^19,39,40^, ${H}_{k}\mathrm{=42}$ Oe, ${\theta }_{k}{\mathrm{=90}}^{\circ }$, ${\phi }_{k}{\mathrm{=5}}^{\circ }$ and film thickness of ${t}_{f}\mathrm{=300}$ nm. The magnetic field is in the film plane, i.e. ${\theta }_{H}{\mathrm{=90}}^{\circ }$, and ${\phi }_{H}$ is varied from 0° up to 360°. For the *V*_*ANE*_ calculation, we assume ${S}_{N}=1.31\times {10}^{-5}$ V/K and the $\Delta T$ varies between 0 and 30 K. To illustrate the results obtained with our routine, Fig. 2 presents the magnetization and thermoelectric voltage calculations for this system.

Figure 2(a), in which we show the magnetization calculation for distinct ${\phi }_{H}$ values, uncovers the expected dependence of the magnetization curves with the orientation between the easy magnetization axis and magnetic field, disclosing all the traditional features of uniaxial magnetic systems^41^. Given that ${\varphi }_{k}{\mathrm{=5}}^{\circ }$, the curve for ${\phi }_{H}={0}^{\circ }$ reveals the signatures of a typical easy magnetization axis. Specifically, the magnetization loop presents normalized remanent magnetization ∼1 and coercive field of around 35 Oe, this latter close to *H*_*k*_. For the magnetization curve at ${\phi }_{H}={90}^{\circ }$ in turn, the normalized remanent magnetization reduces to ∼0.08 and the coercive field to around 2 Oe, values compatible with a hard magnetization axis. Remarkably, the small deviation of ${\phi }_{k}$ yields an hysteretic behavior for ${\phi }_{H}={90}^{\circ }$, giving rise to a typical hard axis behavior with a non-zero coercive field.

Regarding the thermoelectric voltage calculation, Fig. 2(b) shows the *V*_*ANE*_ response, with $\Delta T=27$ K, as a function of the magnetic field for distinct ${\phi }_{H}$ values. Notice the quite-interesting evolution in the shape of the curves as the magnitude and orientation of the field are altered. It is noteworthy that the *V*_*ANE*_ detection is performed with electrical contacts at the ends of the main axis of the film, and here this direction is almost transverse to ${\hat{u}}_{k}$, which defines the orientation of the induced uniaxial magnetic anisotropy. At small ${\phi }_{H}$ values, the *V*_*ANE*_ curves seem to mirror the magnetization loops. This feature is a signature that the magnetization is kept close to the easy magnetization axis, thus favoring the alignment between ${\overrightarrow{E}}_{ANE}$ and the detection direction defined by the electrical contacts. Therefore, *V*_*ANE*_ is directly proportional to the magnitude of the magnetization. On the other hand, as the ${\phi }_{H}$ value increases and the field is not nearby the easy magnetization axis, the *V*_*ANE*_ curves lose the square shape. This fact is a consequence of the competition between two energy terms, the first associated with the Zeeman interaction and the second one related to the induced uniaxial magnetic anisotropy. At the low magnetic field range, the magnetization remains close to the easy magnetization axis, leading to high *V*_*ANE*_ values. However, as the magnetic field increases, the Zeeman energy term dominates, and the magnetization rotates out from the easy axis, following the field. As a result, the ${\overrightarrow{E}}_{ANE}$ component along the detection direction defined by the electrical contacts decreases and *V*_*ANE*_ is drastically reduced.

Figure 2(c) presents the *V*_*ANE*_ values, at $H=\pm 300$ Oe and with $\varDelta T\mathrm{=27}$ K, as a function of ${\phi }_{H}$. At these field values, our system is magnetically saturated, in a sense that the magnetization follows the orientation of the magnetic field. As expected, the curves draw a clear dependence of *V*_*ANE*_ with the sign of the magnetic field and primarily with ${\phi }_{H}$, this latter evidenced by a well-defined cosine shape, as described in Eq. (4).

Further, Fig. 2(d) shows the *V*_*ANE*_ behavior as a function of ${\phi }_{H}$ at $H=+300$ Oe for selected $\varDelta T$ values. In this case, although the amplitude of the curves is altered, the angular dependence is kept constant, also corroborating Eq. (4). At this magnetic saturation state, the maximum *V*_*ANE*_ value is found for ${\phi }_{H}{\mathrm{=0}}^{\circ }$, and corresponds to ${V}_{ANE}^{Smax}$, according to our setup illustrated in Fig. 1(a,b). Moreover, a linear dependence of ${V}_{ANE}^{Smax}$ with $\varDelta T$ is obtained, as we can see in the inset of Fig. 2(d).

After all, the numerical calculations obtained with our theoretical approach provide us an overview of the ANE for a nanostructured system consisting of a film with induced uniaxial magnetic anisotropy. From now, to go beyond, the main challenge to the description of different systems resides in the appropriate writing of the magnetic free energy density.

### Comparison with the experiment

The previous calculations have qualitatively described the main features of the magnetic behavior and thermoelectric voltage response in a film with uniaxial magnetic anisotropy and without stress.

From now on, we investigate the quasi-static magnetic properties and anomalous Nernst effect in a Co_40_Fe_40_B_20_ (from now on called CoFeB) film with thickness of 300 nm grown onto a flexible (Kapton®) substrate (see Methods for details on the film deposition and experiments). In this context, we perform experiments for the sample with and without external stress.

**Figure 3:**
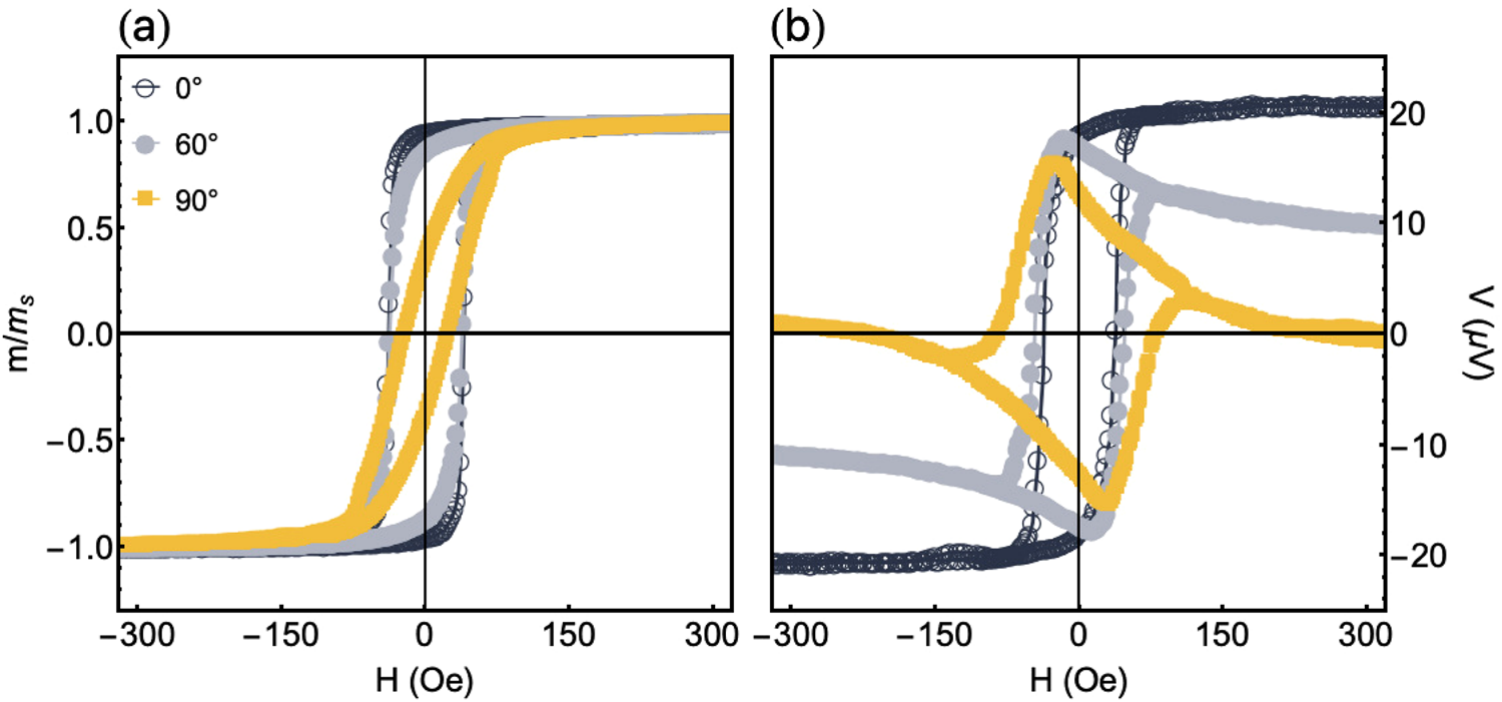
Experimental results of the magnetic response and thermoelectric voltage for the CoFeB film without stress. (**a**) Normalized magnetization curves at selected *φ*_*H*_ values for the CoFeB film. (**b**) Similar plot for the thermoelectric voltage *V*_*ANE*_ measurements performed with $\Delta T=27$ K.

Figure 3 shows experimental results of the magnetic response and the thermoelectric voltage for the CoFeB film without stress. Figure 3(a), in which we present the magnetization response for the film grown onto a flexible substrate as a function of the magnetic field for distinct ${\phi }_{H}$ values, discloses curves that assign the characteristic behavior of a system with uniaxial magnetic anisotropy close to the direction perpendicular to the main axis of the sample. While coercive field of ∼42 Oe and normalized remanent magnetization of ∼1 are found for ${\phi }_{H}={0}^{\circ }$, respective values of ∼30 Oe and ∼0.5 are measured at ${\varphi }_{H}{\mathrm{=90}}^{\circ }$. These values verified for ${\phi }_{H}={90}^{\circ }$ are indicators of magnetic anisotropy dispersion, which is primarily related to the internal stress stored in the film during the growth.

Regarding the thermoelectric voltage results, Fig. 3(b) shows the *V*_*ANE*_ response for the film grown onto a flexible substrate, with $\Delta T\mathrm{=27}$ K, as a function of the magnetic field for distinct ${\phi }_{H}$ values. The experimental *V*_*ANE*_ curves are also in concordance with the numerical calculations presented in Fig. 2(b), reflecting all features of systems with uniaxial magnetic anisotropy and without external stress. Specifically, we find here the evolution in the shape of the curves as the magnitude and orientation of the field are altered. Moreover, at high magnetic field values, when the film is magnetically saturated, we verify the angular dependence of *V*_*ANE*_, having ${V}_{ANE}^{Smax}\approx 20\,\mu$V, and the characteristic reduction of its value to zero as ${\phi }_{H}$ increases to 90°. However, at the low field range, it is noteworthy that a discrepancy between theory and experiment may be found. In particular, as ${\phi }_{H}$ is raised to 90°, *V*_*ANE*_ does not reach the expected maximum of 20 *μ*V found at ${\phi }_{H}={0}^{\circ }$, a value also evidenced numerically in Fig. 2(b). This divergence is primarily associated with the magnetization process at low magnetic fields and to the existence of magnetic domains in the film, a fact that is not taken into account in our microspin modified Stoner-Wohlfarth theoretical approach. Indeed, the magnitude of the magnetization in the experiment is not kept constant at the low-field levels, leading to a reduction of the *V*_*ANE*_ value.

From the experimental results found for the flexible film, shown in Fig. 3 and compared with the calculated ones presented in Fig. 2, we corroborate the angular dependence of the magnetization and thermoelectric voltage curves in films with uniaxial magnetic anisotropy. Going beyond, from now on, we focus our efforts on the magnetic response and thermoelectric voltage in a magnetostrictive system under stress. As aforementioned, CoFeB alloy has high saturation magnetostriction constant, reaching ${\lambda }_{s}\approx +\,30.0\times {10}^{-6}$^19,40^. Therefore, we are able to manipulate here the magnetic properties, i.e. the effective magnetic anisotropy, of our magnetostrictive CoFeB film grown onto a flexible substrate through the application of stress.

**Figure 4:**
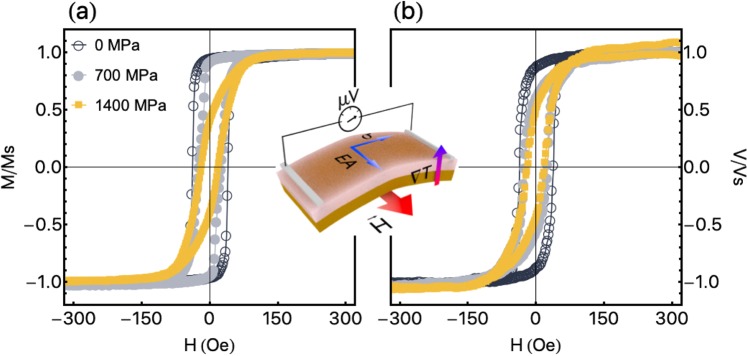
Experimental results of the magnetic response and thermoelectric voltage for the flexible CoFeB film under stress. (**a**) Normalized magnetization curves and (**b**) normalized *V*_*ANE*_ response for the flexible CoFeB film under distinct stress values. The measurements are performed with ${\phi }_{H}={0}^{\circ }$, and bending the sample along its main axis. Moreover, we set $\Delta T=27$ K for the *V*_*ANE*_ acquisition. In the inset, we present the schematic configuration of the sample in the ANE experiment, uncovering the easy magnetization axis (EA) induced during deposition, the orientation of the stress $\sigma$ in the bent film, as well as the directions of the magnetic field, temperature gradient and *V*_*ANE*_ detection.

Figure 4 shows experimental results of the normalized magnetization curves and normalized *V*_*ANE*_ response for the flexible CoFeB film under distinct stress values. The measurements are performed at ${\phi }_{H}={0}^{\circ }$, with the stress along the main axis of the sample (see Methods for details on the stress application). For the positive stress values employed here, ${\lambda }_{s}\sigma  > 0$, and it yields a magnetoelastic anisotropy oriented along the main axis of the sample, i.e. ${\theta }_{\sigma }={90}^{\circ }$ and ${\varphi }_{\sigma }{=90}^{\circ }$.

From the magnetization response shown in Fig. 4(a), we uncover the modifications of the effective magnetic anisotropy with the stress level. Specifically, while the effective anisotropy is well-described by ${\phi }_{{k}_{eff}}$ roughly close to ${\phi }_{k}$ for $\sigma =0$, the changes in the coercive field, remanent magnetization, as well as the own shape of the magnetization curves as the stress level increases are straight consequences of the raise of the magnetoelastic anisotropy contribution to the effective magnetic anisotropy. This raise leads to changes in the orientation ${\hat{u}}_{{k}_{eff}}$ of the effective magnetic anisotropy with the increase of the stress value, thus modifying the whole magnetic behavior and magnetization curves. For $\sigma =700$ MPa, the square curve with smaller coercive field reveals an intermediate magnetic behavior. It arise from an effective magnetic anisotropy that is still roughly close to the direction of the magnetic field at ${\phi }_{H}={0}^{\circ }$, but has a significant component along the main axis of the sample due to the magnetoelastic contribution.

However, for $\sigma =1400$ MPa, the stress is high enough to set the effective magnetic anisotropy axis close to ${\hat{u}}_{\sigma }$, i.e. ${\hat{u}}_{eff}\approx {90}^{\circ }$. Consequently, as expected, the magnetization curve at ${\phi }_{H}={0}^{\circ }$ has fingerprints of a hard magnetization axis, with non-zero values for coercive field and normalized remanent magnetization due to anisotropy dispersion. Therefore, the whole magnetic behavior of the flexible magnetostrictive film under stress is a result of the competition between the induced uniaxial magnetic anisotropy and the magnetoelastic anisotropy contribution. As a consequence, this competition allows us to tailor the effective magnetic anisotropy and, consequently, the anomalous Nernst effect. Remarkably, as we can confirm from Fig. 4(b), modifications in the *V*_*ANE*_ response are already visible at ${\phi }_{H}={0}^{\circ }$, when just the stress is altered in an experimental manner.

**Figure 5:**
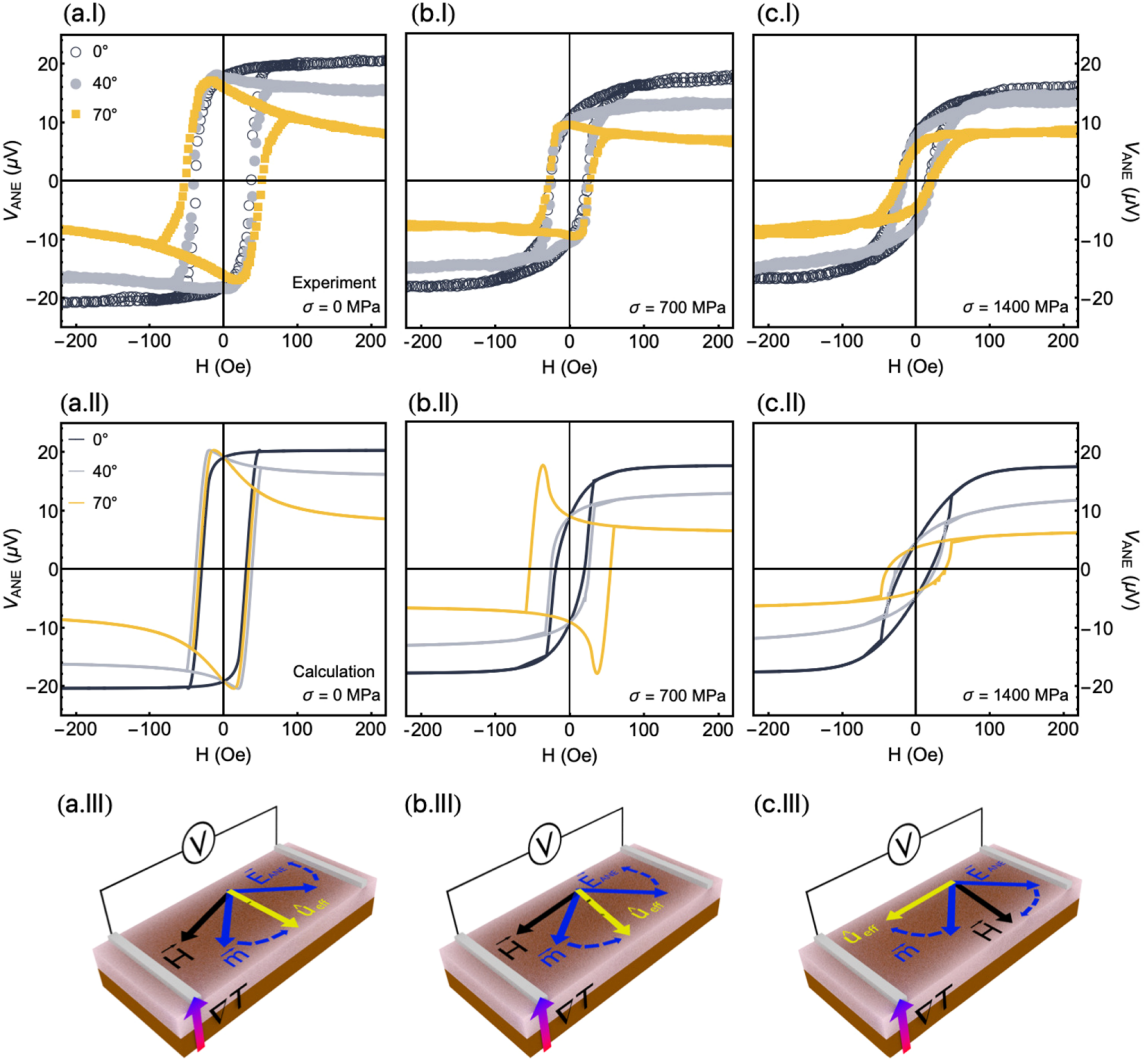
Evolution of the thermoelectric voltage with the magnetic field and stress. (**a**) Experimental results and numerical calculations of the *V*_*ANE*_ response as a function of the magnetic field, for selected *φ*_*H*_ values, for the flexible CoFeB film without stress. Below, illustration showing the orientations of ${\overrightarrow{E}}_{ANE}$, $\overrightarrow{H}$, $\overrightarrow{m}$ and ${\hat{u}}_{eff}$. The dashed lines represent the direction in which $\overrightarrow{m}$ and ${\overrightarrow{E}}_{ANE}$ rotates with decreasing the magnetic field. Similar plots for the flexible CoFeB film submitted to stress values of (**b**) $\sigma =700$ MPa and (**c**) $\sigma =1400$ MPa. We set $\Delta T=27$ K for the *V*_*ANE*_ acquisition. For the numerical calculations, we consider the following parameters: ${m}_{s}=625$ emu/cm^3^, ${\lambda }_{s}=+\,30.0\times {10}^{-6}$, ${H}_{k}=42$ Oe, ${\theta }_{k}={90}^{\circ }$, ${\phi }_{k}={30}^{\circ }$, and ${t}_{f}=300$ nm, with ${S}_{N}=1.31\times {10}^{-5}$ V/K in (**a**), ${S}_{N}=1.14\times {10}^{-5}$ V/K in (**b**), and ${S}_{N}=1.09\times {10}^{-5}$ V/K in (**c**). See the movie in Supplementary Information.

The most striking experimental and theoretical findings here are shown in Fig. 5, which discloses the evolution of the *V*_*ANE*_ response with the ${\phi }_{H}$ and stress *σ* for the CoFeB film grown onto a flexible substrate. The numerical calculations for a film with uniaxial magnetic anisotropy and under stress are performed considering parameters similar to those previously employed in Fig. 2, except for the ${\phi }_{k}$ and *S*_*N*_ values. In this case, ${m}_{s}\mathrm{=625}$ emu/cm^3^ and ${\lambda }_{s}=+\,30.0\times {10}^{-6}$, ${H}_{k}=42$ Oe, ${\theta }_{k}={90}^{\circ }$, ${\phi }_{k}={30}^{\circ }$ and ${t}_{f}=300$ nm. The orientation of ${\hat{u}}_{k}$, set by ${\varphi }_{k}$, is changed in order to mimic the magnetic properties of the flexible film. Further, we consider ${S}_{N}\mathrm{=1.31}\times {10}^{-5}$, $1.14\times {10}^{-5}$ and $1.09\times {10}^{-5}$ V/K (see Methods for details on the *S*_*N*_ estimation) for the calculations with $\sigma =0$, 700, and 1400 MPa, respectively. In particular, the decrease of *S*_*N*_ with $\sigma$ is straightly verified through the reduction of the ${V}_{ANE}^{Smax}$; and we associate it to modifications in the energy relaxation time close of the Fermi level, which can be altered by the stress application^42^. Then, notice the striking agreement between our experiments and numerical calculations, including three important features: the own shape of the *V*_*ANE*_ curves, the ${\phi }_{H}$ dependence of the evolution in the shape of the curves, and amplitude of the *V*_*ANE*_ response at high magnetic fields, i.e. at the magnetic saturation state.

From Fig. 5(a), the *V*_*ANE*_ curves for the film without stress exhibit all the features found for a film with uniaxial magnetic anisotropy, as expected; hence, their interpretation is the very same to that previously reported for Fig. 2(b) and 3(a.II,b.II). With respect to the shape of the curves and its evolution with the orientation of the magnetic field, it is worth observing that the *V*_*ANE*_ response pattern changes with ${\phi }_{H}$ crossing over through ${\phi }_{{k}_{eff}}$. Specifically, keeping in mind that in this case the effective magnetic anisotropy orientation ${\hat{u}}_{{k}_{eff}}$ is the own uniaxial magnetic anisotropy direction ${\hat{u}}_{k}$ with ${\phi }_{k}\approx {30}^{\circ }$, the ${V}_{ANE}$ curves for ${\phi }_{H} < {\phi }_{{k}_{eff}}$ have shape mirroring the corresponding magnetization loops presented in Fig. 4(a). At small ${\phi }_{H}$ values, represented in Fig. 5(a) by the curve for ${\phi }_{H}={0}^{\circ }$, the magnetization is kept close to the magnetic field direction and/or to the easy magnetization axis. It favours the alignment between ${\overrightarrow{E}}_{ANE}$ and the detection direction defined by the electrical contacts, and therefore *V*_*ANE*_ is directly proportional to the magnitude of the magnetization. The *V*_*ANE*_ response for ${\phi }_{H} > {\phi }_{{k}_{eff}}$ in turn, depicted by ${\phi }_{H}={40}^{\circ }$ and 70°, discloses curves with completely different signatures, which lose the square shape. For these cases, at high magnetic field values, the magnetization is aligned with the field and, consequently, *V*_*ANE*_ is drastically reduced; at the low magnetic field range, the magnetization $\overrightarrow{m}$ rotates and remains close to the easy magnetization axis ${\hat{u}}_{eff}$, as we can see in the schematic representation in Fig. 5(a.III), leading to higher *V*_*ANE*_ values.

For the film under $\sigma \approx 700$ MPa, Fig. 5(b) shows curves with a slightly different profile. In this case, the effective magnetic anisotropy is described by an angle ${\phi }_{{k}_{eff}}$ that lies between 40° and 70°, in a sense that the change in the *V*_*ANE*_ response pattern takes place within this angular range. Hence, observe that the *V*_*ANE*_ responses at ${\phi }_{H}={0}^{\circ }$ and 40° have the features of a magnetization curve, just presenting a decrease in the amplitude of the *V*_*ANE*_ signal at the magnetic saturation state, as expected due to the thermoelectric voltage configuration in the experiment. At ${\phi }_{H}={70}^{\circ }$, the shape of the *V*_*ANE*_ curves considerably, suggesting the competition between the Zeeman interaction and the effective magnetic anisotropy. In this case, at high fields, the magnetization follows the magnetic field, leading to a decrease of *V*_*ANE*_. However, as the field decreases, the Zeeman interaction is reduced and the magnetization $\overrightarrow{m}$ turns to ${\hat{u}}_{eff}$. As a consequence, there is the increase in the ${\overrightarrow{E}}_{ANE}$ component along the *V*_*ANE*_ detection direction, as we can see in the schematic representation in Fig. 5(b.III). Obviously, as aforementioned, the discrepancy between experiment and theory at this low-field values is primarily associated with the magnetization process and to the existence of magnetic domains in the film, which is not taken into account in our theoretical approach, as already discussed.

At last, for the film under $\sigma \approx 1400$ MPa shown in Fig. 5(c), the effective magnetic anisotropy lies in an angle ${\phi }_{{k}_{eff}} > {70}^{\circ }$, specifically close to ${\phi }_{{k}_{eff}}\approx {90}^{\circ }$. The presented *V*_*ANE*_ curves have the very same features for all the selected ${\phi }_{H}$ values. At ${\phi }_{H}={0}^{\circ }$, the *V*_*ANE*_ curve mirrors the corresponding magnetization loop shown in Fig. 4(a). As ${\phi }_{H}$ increases, we find the decrease in the amplitude of the *V*_*ANE*_ signal at the magnetic saturation state, as expected. Once the magnetic field decreases, irrespective of ${\phi }_{H}$, the magnetization $\overrightarrow{m}$ rotates to the orientation ${\hat{u}}_{eff}$ of the effective magnetic anisotropy. Given that ${\overrightarrow{E}}_{ANE}$ tends to be almost transverse to the *V*_*ANE*_ detection direction, as we can confirm through the schematic representation in Fig. 5(c.III), a decrease in the *V*_*ANE*_ value is also found at low field values and, as a consequence, the evolution in the shape of the *V*_*ANE*_ curves is not observed here. In particular, for ${\phi }_{H}={90}^{\circ }$, ${V}_{ANE}\approx 0$ for the whole magnetic field range.

**Figure 6:**
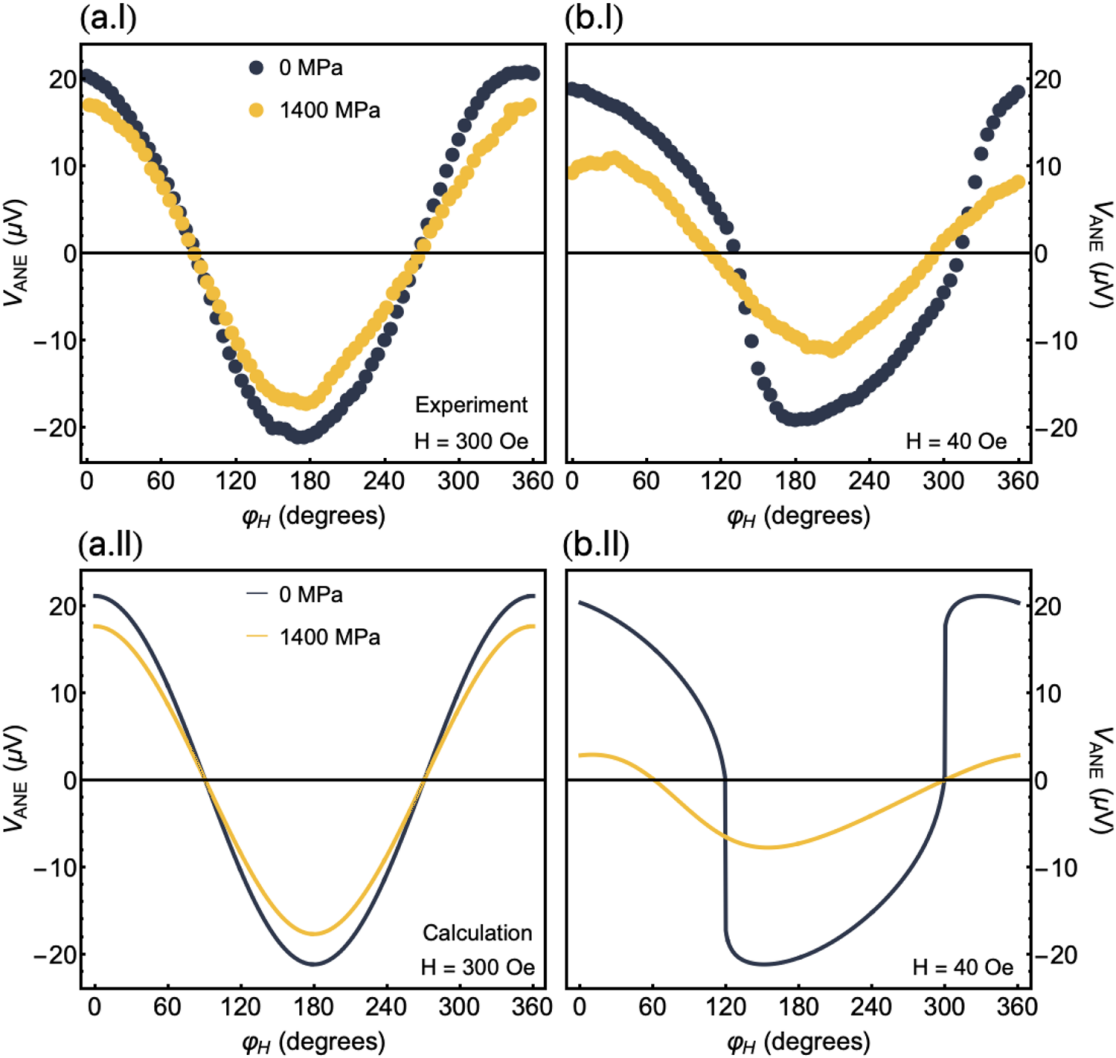
Angular dependence of the thermoelectric voltage. (**a**) Experimental results and numerical calculations for the *V*_*ANE*_, at $H=+\,300$ Oe and with $\varDelta T=27$ K, as a function of *φ*_*H*_ for the flexible CoFeB film under selected stress levels. At this field value, the sample is magnetically saturated. (**b**) Similar plot for the *V*_*ANE*_ at $H=+40$ Oe, where the sample is at a non-saturated state. For the numerical calculations, we consider the very same parameters emloyed in Fig. 5, i.e. ${m}_{s}=625$ emu/cm^3^, ${\lambda }_{s}=+30.0\times {10}^{-6}$, ${H}_{k}=42$ Oe, ${\theta }_{k}={90}^{\circ }$, ${\phi }_{k}={30}^{\circ }$ and ${t}_{f}=300$ nm, with ${S}_{N}=1.31\times {10}^{-5}$ V/K when $\sigma =0$ MPa, and ${S}_{N}=1.09\times {10}^{-5}$ V/K when $\sigma =1400$ MPa.

Finally, looking at the angular dependence of the *V*_*ANE*_ signal amplitude at a given magnetic field, Fig. 6 shows the thermoelectric voltage as a function of ${\phi }_{H}$ for the CoFeB film under selected stress levels. In Fig. 6(a), the sample is magnetically saturated, irrespective on the stress level. As a consequence, the *V*_*ANE*_ response is precisely the one described by Eq. (4), with a well-defined cosine shape. Another important test of consistency of our approach is also given by the *V*_*ANE*_ behavior at non-saturated states. The curves are a result of the competition between the uniaxial magnetic anisotropy and the magnetoelastic anisotropy, leading to considerable changes in the shape and amplitude of the *V*_*ANE*_ response. Notice the striking quantitative agreement between experiment and theory in Fig. 6(b).

Hence, we are able to describe through numerical calculations all the main features of the *V*_*ANE*_ response. Thus, we provide experimental evidence to confirm the validity of the theoretical approach to describe the magnetic properties and anomalous Nernst effect in ferromagnetic magnetostrictive films with uniaxial magnetic anisotropy and submitted to external stress.

## Discussion

In summary, we have performed a theoretical and experimental investigation of the magnetic properties and anomalous Nernst effect in a flexible magnetostrictive film with induced uniaxial magnetic anisotropy and under external stress. Our findings have raised numerous interesting issues on the anomalous Nernst effect in nanostructured magnetic materials. In particular, they have shown how the magnetization behavior and the thermoelectric voltage response evolve with both, the magnetic field and external stress. Hence, by comparing our experiments with numerical calculations, we have elucidated the magnetic properties and thermoelectric voltage and demonstrated the possibility of tailoring the anomalous Nernst effect in a flexible magnetostrictive film. The quantitative agreement between experiment and numerical calculations has provided us evidence to confirm the validity of the theoretical approach to describe the magnetic properties and anomalous Nernst effect in ferromagnetic magnetostrictive films with uniaxial magnetic anisotropy and submitted to external stress. The results place flexible magnetostrictive systems as promising candidates for active elements in functionalized touch electronic devices.

## Methods

### Estimation of Δ*T*_*f*_

The relation between the temperature variation Δ*T* measured experimentally across the sample and the effective temperature variation Δ*T*_*f*_ in the film is given by^43^

6 $$\Delta {T}_{f}=\frac{{t}_{f}{K}_{sub}}{{t}_{sub}{K}_{f}}\Delta T,$$

where *K*_*sub*_ and *t*_*sub*_ are the thermal conductivity and thickness of the substrate, while *K*_*f*_ and *t*_*f*_ are the respective quantities for the ferromagnetic film. Here, we consider ${K}_{sub}=0.12$ W/Km and ${t}_{sub}=0.15$ mm for the flexible substrate (Kapton®), while ${K}_{f}=86.7$ W/Km^44–46^ and ${t}_{f}=300$ nm for our flexible CoFeB film.

### Estimation of *S*_*N*_

**Figure 7:**
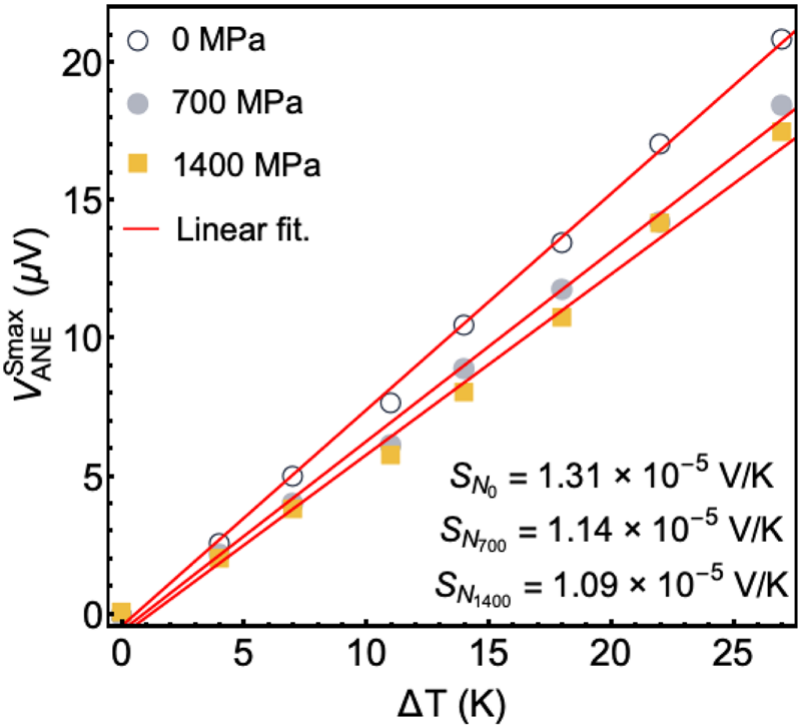
Estimation of the coefficient *S*_*N*_. ${V}_{ANE}^{Smax}$ as a function of Δ*T* for distinct applied stress *σ*. The *S*_*N*_ value is obtained by using Eqs (3) and (6).

We estimate the ${S}_{N}={\lambda }_{N}{\mu }_{\circ }{m}_{s}$ values for each applied stress *σ* level. This coefficient is obtained from the linear fitting of the experimental data of ${V}_{ANE}^{Smax}$ as a function of Δ*T*, as shown in Fig. 7. In particular, $\Delta {T}_{f}$ is calculated by using Eq. (6) and thus *S*_*N*_ is given by Eq. (3). As a result, we assume ${S}_{N}=1.31\times {10}^{-5}$, $1.14\times {10}^{-5}$ and $1.09\times {10}^{-5}$ V/K for the calculations with $\sigma =0$, 700, and 1400 MPa, respectively. Here, it is worth mentioning that the estimated *S*_*N*_ values may be different from the actual ones, given that the measured Δ*T* can include the parasitic contribution due to the interfacial thermal resistance between the sample and the heat sink.

### Sample preparation

We investigate the anomalous Nernst effect in a Co_40_Fe_40_B_20_ (CoFeB) film with thickness of 300 nm grown onto a flexible (Kapton®) substrate. The film is deposited by magnetron sputtering onto a substrate with dimensions of 10 × 6 mm^2^. The deposition process is carried out with the following parameters: base pressure of 5 × 10^−6^ Torr, deposition pressure of 3.0 × 10^−3^ torr with a 99.99% pure Ar at 50 sccm constant flow, and with 50 W set in the DC source. By using these parameters, the deposition rate is 1.86 nm/s. During the deposition, a constant in-plane magnetic field of 1 kOe is applied perpendicularly to the main axis of the substrate in order to induce an uniaxial magnetic anisotropy.

### Magnetization measurements

Quasi-static magnetization curves are obtained within the range between ${\phi }_{H}={0}^{\circ }$ (perpendicular) and ${\phi }_{H}={90}^{\circ }$ (along the main axis of the film), in order to verify the magnetic properties. The curves are acquired at room temperature using a Lake Shore model 7404 vibrating sample magnetometer, with maximum in-plane magnetic field of ±300 Oe.

### Thermoelectric voltage experiments

**Figure 8:**
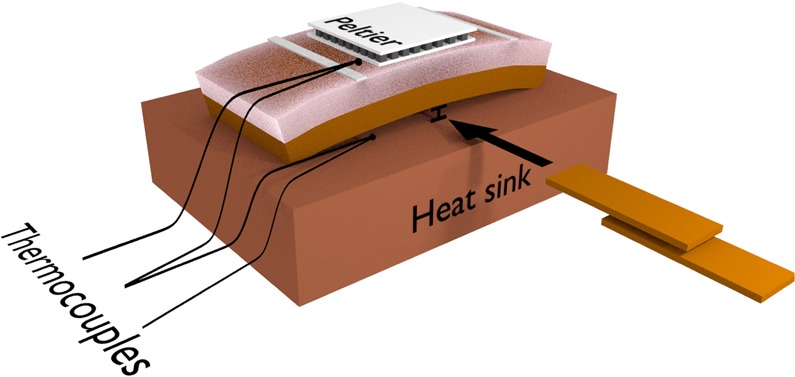
Bending the sample. Kapton strips are place under the film to bend the sample. In particular, micro-thermocouples are used to infer the Δ*T* across the sample (film and Kapton substrate).

We employ a homemade experimental system to measure the thermoelectric voltage associated with the anomalous Nernst effect. In our experimental setup, the temperature gradient ∇*T* is normal to the film plane. A Peltier module is used to heat or cool the top of the sample while the substrate is kept in thermal contact with a heat sink, a copper block at room temperature. The temperature difference $\Delta T$ across the sample (film and Kapton substrate) is measured with a differential micro-thermocouple, depicted in Fig. 8. The magnetic field is kept in the film plane, although its orientation can be modified by varying ${\phi }_{H}$ from 0° up to 360°, having as reference the dashed line indicated in Fig. 1(b). Finally, the *V*_*ANE*_ detection is performed using a nanovoltmeter with electrical contacts at the ends of the main axis of the film, also illustrated in Fig. 1(a,b), whose separation distance is $L=5.0$ mm.

### Stress application

The magnetization measurements and thermoelectric voltage experiments are carried out in a CoFeB film grown onto a flexible substrate. In this context, we perform acquisitions for the sample with and without stress. The stress is applied by bending the sample, along the main axis, during the measurement. In particular, Kapton strips are inserted under the film substrate in order to induce the bending, as illustrated in Fig. 8. The magnitude of the stress *σ* is calculated following the procedure previously employed in refs^36,38^. By bending the flexible substrate, we are able to induce tensile stress in the film, thus modifying the effective magnetic anisotropy^38,47,48^.

## Supplementary information

Supplementary Information
